# Joint Method of Moments (JMoM) and Successive Moment Cancellation (SMC) Multiuser Time Synchronization for ZP-OFDM-Based Waveforms Applicable to Joint Communication and Sensing

**DOI:** 10.3390/s23073660

**Published:** 2023-03-31

**Authors:** Koosha Pourtahmasi Roshandeh, Mostafa Mohammadkarimi, Masoud Ardakani

**Affiliations:** 1 Electrical and Computer Engineering Department, University of Alberta, 116 St. & 85 Ave., Edmonton, AB T6G 2R3, Canada; 2 Faculty of Electrical Engineering, Mathematics and Computer Science, Delft University of Technology, Mekelweg 4, 2628 CD Delft, The Netherlands

**Keywords:** time synchronization, joint method of moments (JMoM) 6G, successive moment cancellation (SMC), coding assisted (CA)-JMoM, CA-SMC, multiuser, OFDM, MIMO, NOMA, second order moment (SoM), zero-padded, non-data-aided, JCAS, ISAC

## Abstract

It has been recently shown that zero padding (ZP)-orthogonal frequency-division multiplexing (OFDM) is a promising candidate for 6G wireless systems requiring joint communication and sensing. In this paper, we consider a multiuser uplink scenario where users are separated in power domain, i.e., non-orthogonal multiple access (NOMA), and use ZP-OFDM signals. The uplink transmission is grant-free and users are allowed to transmit asynchronously. In this setup, we address the problem of time synchronization by estimating the timing offset (TO) of all the users. We propose two non-data-aided (NDA) estimators, i.e., the joint method of moment (JMoM) and the successive moment cancellation (SMC), that employ the periodicity of the second order moment (SoM) of the received samples for TO estimation. Moreover, the coding assisted (CA) version of the proposed estimators, i.e., CA-JMoM and CA-SMC, are developed for the case of short observation samples. We also extend the proposed estimators to multiuser multiple-input multiple-output (MIMO) systems. The effectiveness of the proposed estimators is evaluated in terms of lock-in probability under various practical scenarios. Simulation results show that the JMoM estimator can reach the lock-in probability of one for the moderate range of $E_{b}/N_{0}$ values. While existing NDA TO estimators in the literature either offer low lock-in probability, high computational complexity that prevents them from being employed in MIMO systems, or are designed for single-user scenarios, the proposed estimators in this paper address all of these issues.

## 1. Introduction

Integrating sensing with communication is an inevitable feature in the next generation of wireless networks, i.e., 6G. More specifically, integrated sensing and communication (ISAC), also called joint communication and sensing (JCAS), empowers various radar-based applications such as mobile-based medical imaging and powerful location-aware applications [1]. Integrated communication enhances the radar capability of such devices where each device acts as a node in a distributed radar fusion [2]. On the other hand, the integrated sensing could significantly improve the beam alignment and therefore considerably reduce co-device interference [3]. Due to the enormous potential of JCAS-enabled systems, significant effort, in the past years, from both academia and industry has been put into developing this technology for future wireless networks.

Various waveforms for JCAS systems have been proposed and investigated. However, there is a trade-off between the communication and sensing capability of any proposed technique [4,5,6,7]. The reason is that random signals are required for communication to convey information; however, deterministic signals are employed for sensing. For example, frequency-modulated continuous wave signals (Frequency-modulated continuous wave signals are used in radar applications) in combination with quadrature amplitude modulation (QAM) or frequency shift keying offer high sensing abilities with relatively simple transceiver design but suffer from low spectral efficiency [8,9,10]. On the other hand, 5G’s cyclic prefix (CP)-OFDM signals provide high spectral efficiency but exhibit relatively low sensing capabilities compared to their counterparts, frequency-modulated continuous wave signals [3].

Recently, ZP-OFDM-based waveforms are shown to be promising candidates for ISAC because they offer a trade-off between spectral efficiency for communication and sensing capabilities [3]. More specifically, ZP-OFDM-based waveforms can operate in half-duplex instead of CP-OFDM systems that can perform JCAS only in full-duplex mode. This is important because practical full-duplex implementations consume a lot of energy and are costly. Moreover, self-interference in full-duplex scenarios such as in CP-OFDM systems is of great concern as the received power of the echoed signal is orders of magnitude less than that of the transmitted signal. This is due to the fact that the echoed signal travels twice the distance to the target and its power decays with the fourth power of the distance. The problem gets worse given that practical full-duplex implementations offer limited self-interference isolation between the transmitted signal and the received signal. On the contrary, ZP-OFDM can take advantage of the silent period, i.e., guard interval, in the transmission signal in order to receive the echo signals and perform sensing tasks. This eliminates the need for costly full-duplex implementation. In addition, ZP-OFDM based waveforms offer a higher peak power compared to the CP-OFDM based waveforms, which makes them more interesting for sensing.

In terms of communication capabilities and features, also, ZP-OFDM offers various advantages over CP-OFDM [11] such as enabling finite impulse response equalization of the channels irrespective of channel nulls and improving BER through guaranteeing symbol recovery regardless of the channel zeros. Moreover, ZP-OFDM makes channel tracking and estimation simpler and exhibits higher power efficiency compared to CP-OFDM as it does not resend cyclic data samples [11].

Despite all these benefits, there are practical issues that need to be solved in order to make ZP-OFDM a viable solution for JCAS. One such a problem is the time synchronization. Given the massive number of antennas, connected devices, and subcarriers in 6G systems, the pilot transmission overhead becomes a bottleneck for both extremely high spectral efficiency and ultralow latency requirements of future wireless systems [12]. Various methods are therefore proposed to reduce or ideally remove the pilots [12,13,14]. However, reducing (or removing) the pilots makes the time synchronization task in OFDM systems significantly more challenging. This becomes a crucial problem specifically for JCAS zero-padding-based OFDM waveform candidates, e.g., ZP Dual Index Trimode OFDM-IM [15], ZP-OTFS [16,17], and RP-OTFSM [18], for 6G, compared to their counterpart, i.e., CP-OFDM, due to the lack of CP.

Estimating the timing offset for time synchronization without the need for pilots and preamble is called NDA time synchronization. NDA or semi-NDA timing offset estimation are promising solutions for ISAC since the spectral efficiency of the communication increases in the absence or reduction of pilots and preamble. NDA timing offset estimation methods for ZP-based OFDM waveforms, such as ZP Dual Index Trimode OFDM-IM [15], ZP-OTFS [16,17], and RP-OTFSM [18], mostly rely on heuristic techniques such as the one in [19] where a transition metric (TM) is defined. In such metrics, usually, the ratio of the power of a window of nonzero transmitted samples over the power of a window of zero samples (which are received as noise samples at the receiver) is calculated. Then, the point where this ratio is maximized is considered as the timing offset. Such techniques, though simple, have a poor probability of correct estimation, called lock-in probability. On the other hand, a mathematically heavy approach via maximum likelihood (ML) technique was proposed in [20]. However, the approach in [20] is highly complex, which hinders its implementation in many practical scenarios where the user has limited computational capacity, e.g., mobile users and sensors. Moreover, the proposed method in [20] cannot be used for signal-to-noise ratio (SNR)s less than 5 dB due to the accumulation of numerical errors. In an attempt to address these issues, authors in [21] proposed two methods based on the method of moments. In such techniques, the timing offset is estimated by equating the theoretical moments of the received samples and their natural moments. The lowest possible moment order was used by the authors in [21] in order to keep the computational complexity as low as possible. The advantage of this technique besides its low complexity compared to that of [20] is its ability to be implemented for all SNR ranges, especially very low SNRs. However, the scenario considered in [21] is for single-user transmission, and does not address when there are multiple users and the users are separated in power domain, i.e., NOMA. NOMA is a major multiple access candidate for 6G wireless systems in order to provide the required data rates for ever-increasing number of connected devices [22,23,24,25].

In this paper, we consider a multiuser uplink scenario where users are separated in power domain, i.e., NOMA. ZP-OFDM signal is used for data transmission. A grant-free uplink transmission scheme is considered where users transmit asynchronously. The problem of time synchronization is investigated by estimating the timing offset (TO) of all the users. We propose two NDA TO estimators, i.e., the JMoM and the SMC, that utilize the periodicity of the SoM of the received samples in order to estimate the TO. Furthermore, we develope the CA version of the proposed estimators, called CA-JMoM and CA-SMC, for the scenarios where the number of observation samples are short. The proposed estimators are then extended to MIMO systems. Finally, the performance of the proposed estimators, in terms of lock-in probability, is evaluated under different practical scenarios.

## 2. Materials and Methods

In this section, we first discuss the system model for NDA time synchronization for ZP-OFDM. Then, two NDA estimators, i.e., JMoM and SMC estimators are proposed.

### 2.1. System Model

We consider that users $u_{1},u_{2},\ldots ,u_{U}$ asynchronously communicate with a single base station (BS) via ZP-OFDM technique through doubly selective fading channels. It is assumed that both users and the BS can be mobile, and there is no restrictions on the relative radial velocity of the BS and the users. That is, the users can move with ultrafast speeds while communicating with the BS. Let $p_{1},p_{2},\ldots ,p_{U}$ and $\tau_{1},\tau_{2},\ldots ,\tau_{U}$ denote the transmit power and the TO between the *U* users and the BS, respectively. In this paper, we consider TO estimation in the sample level, i.e., $\tau_{i}=d_{i}$, i=1,2,…,U, where $d_{i}\in N$. The fractional part of the TO appears as phase offset at each subcarrier. Hence, its effect is compensated when carrier frequency offset is estimated [26]. Moreover, sample level synchronization offers high accurate sensing for wide-band systems. It is assumed that the BS does not have prior knowledge on the pilots and preambles inserted in the ZP-OFDM signals of the users. Hence, the BS needs to employ NDA estimators for time synchronization and channel estimation to be able to decode the ZP-OFDM symbols.

Let ${\left\{x_{n,k}^{\left(i\right)}\right\}}_{k=0}^{n_{x}-1},\forall i\in \left\{1,2,\cdots ,U\right\}$ denote the $n_{x}$ complex valued modulated symbols to be transmitted from user $u_{i}$ to the BS with the average of power $p_{i}=E\{|x_{n,k}^{\left(i\right)}{|}^{2}\}=\sigma_{x_{i}}^{2}$. The subscript *n* denotes the *n*th OFDM symbol, and *k* denotes the *k*th sample of an OFDM symbol. The *n*th baseband OFDM symbol of user $u_{i}$ is expressed as [27]

(1) $$\begin{array}{c} x_{n}^{\left(i\right)}\left(t\right)=\sum_{k=0}^{n_{x-1}}x_{n,k}^{\left(i\right)}e^{\frac{j2\pi kt}{T_{x}}},\;\;\;\;\;\;\;\;\;\;\;0\leq t\leq T_{x}, \end{array}$$

where $T_{x}$ denotes the OFDM symbol duration before zero-padding. Each OFDM symbol is then zero-padded in order to mitigate the effect of inter symbol interference (ISI). Therefore, one can write the final zero-padded *n*th OFDM symbol as

(2) $$\begin{array}{c} s_{n}^{\left(i\right)}\left(t\right)=\left\{\begin{array}{c} x_{n}^{\left(i\right)}\left(t\right)\;\;\;\;\;\;\;\;\;\;\;\;0\leq t<T_{x}\\ 0\;\;\;\;\;\;\;\;\;\;\;\;\;\;\;\;\;\;\;\;\;\;\;\;T_{x}\leq t<T_{x}+T_{z}. \end{array}\right. \end{array}$$

The signal described in Equation (2) then goes through a multipath wireless channel. The baseband impulse response of the channel can be written as

(3) $$\begin{array}{c} h\left(t,\tau \right)=\sum_{l}\alpha_{l}\left(t\right)\delta \left(\tau -\tau_{l}\right), \end{array}$$

where $\alpha_{l}\left(t\right)$ is a complex number and $\tau_{l}$ denotes the *l*th channel tap’s delay. The effect of transmit and receive filters are captured in h(t,τ). It should be noted that *t* in Equation (3) captures the time selectivity of the channel, i.e., time-varying channel. Moreover, different $\tau_{l}$ values express the frequency selectivity of the channel, i.e., frequency-varying. Hence, the channel is considered to be doubly selective.

Let $\tau_{d}$ denote the delay spread of the multipath channel, where $E\{|\alpha_{l}\left(t\right){|}^{2}\}=0$ for $\tau_{l}>\tau_{d}$. The length of the zero-padding guard interval is then chosen such that $T_{z}\geq \tau_{d}$. The sampling time for the received signal at the BS is assumed to be $T_{\mathrm{sa}}=T_{x}/n_{x}$. With the assumption of perfect time synchronization, the complex received basband signal of user $u_{i}$ at the BS can be written as

(4) $$\begin{array}{c} v_{n}^{\left(i\right)}\left[k\right]=\sum_{l=0}^{n_{h}-1}h\left[nn_{s}+k;l\right]s_{n}^{\left(i\right)}\left[k-l\right], \end{array}$$

where

(5) $$\begin{array}{cc} \; & n_{s}=\left\lfloor T_{s}/T_{\mathrm{sa}}\right\rfloor =\left\lfloor \left(T_{x}+T_{z}\right)/T_{\mathrm{sa}}\right\rfloor \end{array}$$

(6) $$\begin{array}{cc} \; & s_{n}\left[k\right]≜s_{n}\left(kT_{\mathrm{sa}}\right),\;\;\;\mathrm{for}\;\;k=0,1,\ldots ,n_{s}-1, \end{array}$$

$n_{h}≜\left\lfloor \tau_{d}/T_{\mathrm{sa}}\right\rfloor$ denotes he number of channel taps, and ⌊.⌋ denotes the floor function.

Wide-sense stationary uncorrelated scattering (WSSUS) is assumed for the channel model, and the channel taps are assumed to be independent random variables. We assume the channel coefficients follow zero-mean complex Gaussian distribution, i.e., Rayleigh fading is considered, and the power delay profile (PDP) of the channel is modeled as

(7) $$\begin{array}{c} E\{h\left[k_{1},l\right]h^{*}\left[k_{2},l-m\right]\}=\sigma_{h_{l}}^{2}R\left[k1-k2\right]\delta \left[m\right],\;\;\;\;\;\;\;\;l=0,1,\ldots ,n_{h}-1, \end{array}$$

where

(8) $$\begin{array}{c} \sigma_{h_{l}}^{2}={E\{|h\left[k,l\right]|}^{2}\}, \end{array}$$

and R[k1−k2] is an arbitrary function with R[0]=1. As the relative speed of the transmitter and the receiver increases, $R[k_{1}-k_{2}]$ approaches $\delta [k_{1}-k_{2}]$. In this paper, we consider ultrafast speeds; thus, the PDP of the channel is modeled as

(9) $$\begin{array}{c} E\{h\left[k,l\right]h^{*}\left[k,l-m\right]\}=\sigma_{h_{l}}^{2}\delta \left[m\right],\;\;\;\;\;\;\;\;l=0,1,\ldots ,n_{h}-1. \end{array}$$

We assume that the PDP of the channel is priori known at the BS and has already been estimated during channel sounding, where a known signal is transmitted and the power of the received signal at the receiver is measured and then averaged.

Let $n_{s}≜n_{x}+n_{z}$ denote the number of samples per ZP-OFDM symbols, where $n_{z}$ denotes the number of zeros padded to each OFDM symbol and is given by $n_{z}≜\left\lfloor T_{z}/T_{\mathrm{sa}}\right\rfloor$. In the absence of TO, by using (4) and considering the additive white Gaussian noise (AWGN) at the receiver, we can write the received signal at the BS in vector form as

(10) $$\begin{array}{c} y_{n}=\left\{\begin{array}{cc} \sum_{i=1}^{U}v_{n}^{\left(i\right)}+w_{n}≜\sum_{i=1}^{U}H^{\left(i\right)}s_{n}^{\left(i\right)}+w_{n}, & \;\;\;\;\;\;\;\;\;\;\;\;n\geq 0\\ w_{n}, & \;\;\;\;\;\;\;\;\;\;\;\;n<0, \end{array}\right. \end{array}$$

where

(11) $$s_{n}^{\left(i\right)}≜\left[\begin{array}{c} \;\;\;\;\;\;s_{n}^{\left(i\right)}\left[0\right]\\ \;\;\;\;\;\;s_{n}^{\left(i\right)}\left[1\right]\\ \;\;\;\;\;\;\;\;\;\;\vdots \\ s_{n}^{\left(i\right)}\left[n_{s}-1\right] \end{array}\right]=\left(\begin{array}{c} x_{n}^{\left(i\right)}\left(0\right)\\ \vdots \\ x_{n}^{\left(i\right)}\left(\left(n_{x}-1\right)T_{\mathrm{sa}}\right)\\ 0\\ \vdots \\ 0 \end{array}\right),$$

(12a) $$\begin{array}{cc} y_{n}^{\left(i\right)} & ≜[y_{n}^{\left(i\right)}\left[0\right],\;y_{n}^{\left(i\right)}\left[1\right],\;\ldots \;y_{n}^{\left(i\right)}\left[n_{s}-1\right]{]}^{T}, \end{array}$$

(12b) $$\begin{array}{cc} w_{n} & ≜[w_{n}\left[0\right],\;w_{n}\left[1\right],\;\ldots \;w_{n}\left[n_{s}-1\right]{]}^{T}\;∼\mathrm{CN}\left(0,\sigma_{w}^{2}I_{n_{s}}\right), \end{array}$$

(12c) $$\begin{array}{cc} v_{n}^{\left(i\right)} & ≜[v_{n}^{\left(i\right)}\left[0\right],\;v_{n}^{\left(i\right)}\left[1\right],\ldots v_{n}^{\left(i\right)}\left[n_{s}-1\right]{]}^{T}=H^{\left(i\right)}s_{n}^{\left(i\right)}, \end{array}$$

and $I_{m}$ and $\sigma_{w}^{2}$ denote the m×m identity matrix and the variance of the noise, respectively. The convolution of the signal and channel taps are expressed via the multiplication of the signal, $s_{n}^{\left(i\right)},$ and an $n_{s}\times n_{s}$ Toeplitz channel matrix $H^{\left(i\right)}$, where its *i*th ($0\leq i\leq n_{s}-1$) column is ${\left[0_{i}^{T}\;h\left[nn_{s}+i-1,0\right]\;h\left[nn_{s}+i-1,1\right]\;\ldots \;h\left[nn_{s}+i-1,n_{h}-1\right]\;0_{n_{s}-n_{h}-i}^{T}\right]}^{T}$.

### 2.2. JMoM TO Estimator

Let us write the vector of the received samples as

(13) $$\begin{array}{cc} y & ≜[y_{1}\left[0\right],\;y_{1}\left[1\right],\;\ldots \;y_{1}\left[n_{s}-1\right],\;\ldots \;,y_{\left\lfloor \frac{M}{n_{s}}\right\rfloor }\left[0\right]\;,\;y_{\left\lfloor \frac{M}{n_{s}}\right\rfloor }\left[1\right],\;\ldots \;y_{\left\lfloor \frac{M}{n_{s}}\right\rfloor }\left[n_{s}-1\right]{]}^{T}, \end{array}$$

where *M* denotes the total number of received samples and is considered to be a multiple of $n_{s}$ for the simplicity of notation (arbitrary values can be considered for *M*). Now, by using (10), we can write the received vector of length *M* as

(14) $$\begin{array}{cc} y & =\sum_{i=1}^{U}v^{\left(i\right)}+w, \end{array}$$

where

(15) $$\begin{array}{cc} v^{\left(i\right)} & ≜[v_{1}^{\left(i\right)}\left[0\right],\;v_{1}^{\left(i\right)}\left[1\right]\;\ldots \;v_{1}^{\left(i\right)}\left[n_{s}-1\right],\;\ldots \;,\;v_{\left\lfloor \frac{M}{n_{s}}\right\rfloor }^{\left(i\right)}\left[0\right],\;v_{\left\lfloor \frac{M}{n_{s}}\right\rfloor }^{\left(i\right)}\left[1\right],\;\ldots \;v_{\left\lfloor \frac{M}{n_{s}}\right\rfloor }^{\left(i\right)}\left[n_{s}-1\right]{]}^{T}\\ \; & =[H^{\left(i\right)}s_{1}^{\left(i\right)},\;\ldots \;,H^{\left(i\right)}s_{\left\lfloor \frac{M}{n_{s}}\right\rfloor }^{\left(i\right)}{]}^{T}, \end{array}$$

and

(16) $$\begin{array}{cc} w & ≜[w_{1}\left[0\right],\;w_{1}\left[1\right],\;\ldots \;w_{1}\left[n_{s}-1\right],\;\ldots \;,\;w_{\left\lfloor \frac{M}{n_{s}}\right\rfloor }\left[0\right],\;w_{\left\lfloor \frac{M}{n_{s}}\right\rfloor }\left[1\right],\;\ldots \;w_{\left\lfloor \frac{M}{n_{s}}\right\rfloor }\left[n_{s}-1\right]{]}^{T}. \end{array}$$

Let $H_{d_{i}}$ denote the hypothesis that the TO of the user $u_{i}$ with respect to the BS reference clock is $d_{i}\in \left\{-d_{\max},\ldots ,-1,0,1,\ldots ,d_{\max}\right\}$ where $1\leq d_{\max}\leq n_{s}-1$. Also, the hypothesis $H_{d_{1},\ldots ,d_{U}}$ denotes that the TOs of the users $u_{1},u_{2},\ldots ,u_{U}$ with respect to the BS reference clock are $d_{1},d_{2},\ldots ,d_{U}$, respectively. Given hypothesis $H_{d_{1},\ldots ,d_{U}}$, the received vector at the BS can be written as

(17) $$\begin{array}{cc} y|H_{d_{1},\ldots ,d_{U}} & =\sum_{i=1}^{U}v^{\left(i\right)}|H_{d_{i}}+w, \end{array}$$

where

(18) $$\begin{array}{cc} v^{\left(i\right)}|H_{d_{i}\geq 0} & =[0_{d_{i}}^{T},v_{1}^{\left(i\right)}\left[0\right],\;v_{1}^{\left(i\right)}\left[1\right],\;\ldots \;,v_{1}^{\left(i\right)}\left[n_{s}-1\right],\;\ldots \;,\;v_{\left\lfloor \frac{M}{n_{s}}\right\rfloor }^{\left(i\right)}\left[0\right],\;\ldots \;,v_{\left\lfloor \frac{M}{n_{s}}\right\rfloor }^{\left(i\right)}\left[n_{s}-1\right]{]}^{T}, \end{array}$$

with $0_{d_{i}}$ as the vector of all zeros of length $d_{i}\in \left\{0,1,\ldots ,d_{\max}\right\}$, and

(19) $$\begin{array}{cc} v^{\left(i\right)}|H_{d_{i}<0} & =[v_{1}^{\left(i\right)}\left[\right|d_{i}|],\;v_{1}^{\left(i\right)}\left[\right|d_{i}\left|+1\right],\;\ldots \;,v_{1}^{\left(i\right)}\left[n_{s}-1\right],\;\ldots \;,\;v_{\left\lfloor \frac{M}{n_{s}}\right\rfloor }^{\left(i\right)}\left[0\right],\;\ldots \;,v_{\left\lfloor \frac{M}{n_{s}}\right\rfloor }^{\left(i\right)}\left[n_{s}-1\right]{]}^{T} \end{array}$$

for  $-d_{\max}\leq d_{i}<0$. We allow the TO $d_{i}$ to take both negative and positive values. A positive value for $d_{i}$ means that the BS receives signal before the reception of the user $u_{i}$ signal. On the other hand, a negative TO value means that the BS has missed the first $d_{i}$ samples of the user $u_{i}$.

We assume that the theoretical SoM of $v_{n}^{\left(i\right)}\left[j\right]$, i=1,2,…,U, $j=0,1,\ldots ,n_{s}-1$, and n≥0, in (18) and (19) given hypothesis $H_{d_{i}\geq 0}$ and $H_{d_{i}<0}$, i.e.,

(20a) $$\begin{array}{cc} \sigma_{v^{\left(i\right)}|H_{d_{i}\geq 0}}^{2} & =\mathrm{diag}(E\{v^{\left(i\right)}\left|H_{d_{i}\geq 0}(v^{\left(i\right)}\right|H_{d_{i}\geq 0}{)}^{H}\}), \end{array}$$

(20b) $$\begin{array}{cc} \sigma_{v^{\left(i\right)}|H_{d_{i}<0}}^{2} & =\mathrm{diag}(E\{v^{\left(i\right)}\left|H_{d_{i}<0}(v^{\left(i\right)}\right|H_{d_{i}<0}{)}^{H}\}), \end{array}$$

are known at the BS. In (20a) and (20b), diag(·) and ${\left(\cdot \right)}^{H}$ denote a vector consists of the diagonal elements of a matrix and the Hermitian of a matrix, respectively.

The authors have analytically obtained the theoretical conditional SoM vectors $\sigma_{v^{\left(i\right)}|H_{d_{i}\geq 0}}^{2}$ and $\sigma_{v^{\left(i\right)}|H_{d_{i}<0}}^{2}$, in Theorem 1 in [21]. By using the vector of theoretical SoMs [21] and the squared of the absolute values (SAV) of the received samples, we can express the problem of multiuser TO estimation for users $u_{1},u_{2},\ldots ,u_{U}$ as

(21) $$\begin{array}{cc} {\hat{d}}_{1},\cdots ,{\hat{d}}_{U} & =\underset{-d_{\max}\leq d_{1},\cdots ,d_{U}\leq d_{\max}}{\mathrm{argmin}}∥P_{y}-\sigma_{y|H_{d_{1},\cdots ,d_{U}}}^{2}∥\\ \; & =\underset{-d_{\max}\leq d_{1},\cdots ,d_{U}\leq d_{\max}}{\mathrm{argmin}}∥P_{y}-\sum_{i=1}^{U}\sigma_{v^{\left(i\right)}|H_{d_{i}}}^{2}-\sigma_{w}^{2}1_{M}∥, \end{array}$$

where $∥.∥$ and $1_{M}$ denote norm two, and a vector of ones with length *M*, respectively, ${\hat{d}}_{1},\cdots ,{\hat{d}}_{U}$ denote the estimated TO of the users $u_{1},u_{2},\ldots ,u_{U}$,

(22) $$\begin{array}{cc} \; & P_{y}=[|y_{1}\left[0\right]{|}^{2},\;|y_{1}\left[1\right]{|}^{2},\;\ldots \;|y_{1}\left[n_{s}-1\right]{|}^{2},\;\ldots \;,\;|y_{\left\lfloor \frac{M}{n_{s}}\right\rfloor }\left[0\right]{|}^{2},\;|y_{\left\lfloor \frac{M}{n_{s}}\right\rfloor }\left[1\right]{|}^{2},\;\ldots \;{\left|y_{\left\lfloor \frac{M}{n_{s}}\right\rfloor }\left[n_{s}-1\right]\right|}^{2}{]}^{T}, \end{array}$$

and

(23) $$\begin{array}{c} \sigma_{v^{\left(i\right)}|H_{d_{i}}}^{2}=\sigma_{v^{\left(i\right)}|H_{d_{i}\geq 0}}^{2}I_{R^{+}}\left(d_{i}\right)+\sigma_{v^{\left(i\right)}|H_{d_{i}<0}}^{2}I_{R^{-}}\left(d_{i}\right), \end{array}$$

where $I_{R^{+}}\left(d_{i}\right)$ denotes the indicator function and $R^{+}$ and $R^{-}$ denote the ranges $\left[0,d_{\max}\right]$ and $\left[-d_{\max},0\right)$, respectively.

### 2.3. Successive Moment Cancellation (SMC) TO Estimator

The proposed JMoM multiuser TO estimator suffers from huge computational complexity as the number of users increases. In this subsection, we propose the low-complexity SMC multiuser TO estimator inspired by the successive interference cancelation (SIC) algorithm [28]. The main idea behind the proposed SMC is to first estimate the TO of the user with the largest average theoretical SoM, i.e., $\left|\right|\sigma_{v^{\left(i\right)}|H_{0}}^{2}\left|\right|/M$ by using the Method of Moments (MoM). Then, by subtracting the theoretical conditional SoMs of the first user and the vector of noise variance from the SAV of the received samples, the TO of the next user with the largest average theoretical SoM is estimated. This procedure, without subtracting the vector of noise variance, continues until the TO of the last user is estimated. While in power NOMA for communications, difference in the power of the users is a necessary condition, power NOMA synchronization is feasible for user with equal transmit power because for the NOMA synchronization only the level of shift in the sequence of a prior known SoM is unknown. The proposed SMC multiuser TO estimator is summarized in Algorithm 1. In Algorithm 1, δ[·] is the Kronecker delta function.
**Algorithm 1** SMC1:**Initialization: **$\hat{d}\leftarrow 0_{U}^{T},\mathrm{calculate}\;\sigma_{v^{\left(i\right)}|H_{d_{i}}}^{2}$ in (23) and $P_{y}$ in (22)2:**Preprocessing:** Sort users  and their $\sigma_{v^{\left(i\right)}|H_{d_{i}}}^{2}$ in descending order of $\left|\right|\sigma_{v^{\left(i\right)}|H_{0}}^{2}\left|\right|/M$3:i←U4:**while **i>0** do**5:    k←U−i+16:    i←i−17:    $L_{\min}\leftarrow \mathrm{Inf}$8:    **for** $d:=-d_{\max}:d_{\max}$ **do**9:        $L\leftarrow ∥P_{y}-\sigma_{v^{\left(k\right)}|H_{d}}^{2}-\sigma_{w}^{2}1_{M}\delta \left[U-i-1\right]∥$10:       **if** $L<L_{\min}$ **then**11:           $\hat{d}\left[k\right]\leftarrow d$12:           $L_{\min}\leftarrow L$13:    $P_{y}\leftarrow P_{y}-\sigma_{v^{\left(k\right)}|H_{\hat{d}\left[k\right]}}^{2}-\sigma_{w}^{2}1_{M}\delta \left[U-i-1\right]$14:**return **$\hat{d}$

### 2.4. Coding-Assisted (CA) Estimator for Fast TO Estimation

In some highly resource-limited scenarios, the proposed JMoM and the SMC multiusr TO estimators may fall short to achieve very high lock-in probabilities. One such scenario is when the users not only use single antenna for data transmission but also have very limited memory. In such scenario, a sufficient number of OFDM symbols can not be loaded into the memory and used for TO estimation in order to achieve the desired accuracy. Hence, the performance of the proposed JMoM and SMC estimators degrades. In order to address such scenarios, we propose the idea of CA TO estimator that can be employed in combination with JMoM and SMC algorithms for performance improvement.

Various performance metrics are used for evaluating different TO estimators. The mean-squared error (MSE) and lock-in probability are two main metrics that are used in the literature. Lock-in probability, i.e., $P_{l}$, is the strictest measure as estimating the TO off by only one sample before or after the actual TO value counts as an error. On the other hand, MSE is the most lenient measure because while it gives an overview of the overall performance, it hides various important information such as how many times the estimator correctly estimated the actual TO value or exactly how far off are the estimated TO values from the actual value. For example, an estimator could always wrongly estimate the TO but have a lower MSE compared to an estimator which correctly estimates the TO for certain times but the wrongly estimated values vary far off from the actual TO value. Hence, we define a new metric where up to maximum $n_{\epsilon }$ sample error, i.e., $|{\hat{d}}_{i}-d_{i}|\leq n_{\epsilon }\;\mathrm{for}\;i=1,\cdots ,U$, is considered as lock-in region (LR). The probability of correct multiuser time synchronization in the LR is given by

(24) $$\begin{array}{c} P_{\mathrm{lr}}\left(n_{\epsilon }\right)=\overset{U}{\underset{i=1}{⋂}}P(|{\hat{d}}_{i}-d_{i}\left|\leq n_{\epsilon }\right), \end{array}$$

where lr stands for lock-in region, ⋂ is the intersection operator, and $P(|{\hat{d}}_{i}-d_{i}|\leq n_{\epsilon })$ is the probability of correct synchronization for user $u_{i}$ in its lock-in region. It is obvious that $P_{l}=P_{\mathrm{lr}}\left(0\right)$.

In the CA-JMoM and the CA-SMC multiuser TO estimators, a forward error correction (FEC), via low-density parity-check code (LDPC), cyclic redundancy check (CRC), etc [29], is conducted for $n_{\epsilon }^{U}$ TO combinations in the lock-in region, and estimated TO vector is the one that passes the parity check and achieves the lowest TO MSE.The proposed SMC multiuser TO estimator is summarized in Algorithm  2.
**Algorithm 2** CA-SMC** Step 1:**1:**Initialization: **$\hat{d}\leftarrow 0_{U}^{T},\mathrm{calculate}\;\sigma_{v^{\left(i\right)}|H_{d_{i}}}^{2}$ in (23) and $P_{y}$ in (22)2:**Preprocessing:** Sort users  and their $\sigma_{v^{\left(i\right)}|H_{d_{i}}}^{2}$ in descending order of $\left|\right|\sigma_{v^{\left(i\right)}|H_{0}}^{2}\left|\right|/M$3:i←U4:**while **i>0** do**5:    k←U−i−16:    i←i−17:    $L_{\min}\leftarrow \mathrm{Inf}$8:    **for** $d:=-d_{\max}:d_{\max}$ **do**9:        $L\leftarrow ∥P_{y}-\sigma_{v^{\left(k\right)}|H_{d}}^{2}-\sigma_{w}^{2}1_{M}\delta \left[U-i-1\right]∥$10:        **if** $L<L_{\min}$ **then**11:           $\hat{d}\left[k\right]\leftarrow d$12:           $L_{\min}\leftarrow L$13:    $P_{y}\leftarrow P_{y}-\sigma_{v^{\left(k\right)}|H_{\hat{d}\left[k\right]}}^{2}-\sigma_{w}^{2}1_{M}\delta \left[U-i-1\right]$** Step 2:**14:Derive $\mathrm{LR}=\{d\in Z^{U}\;\;|\;\;|{\hat{d}}_{i}-d_{i}|\leq n_{\epsilon },\;\forall i\in \left\{1,\ldots ,U\right\}\;\;\}$ based on $n_{\epsilon }$ and $\hat{d}$15:Derive ${\mathrm{LR}}_{\mathrm{selected}}=\left\{d\in \mathrm{LR}\;\;|\;\;\mathrm{pass}\;\mathrm{parity}\;\mathrm{check}\right\}$16:$F_{\min}\leftarrow \mathrm{Inf}$17:**for **$d\in {\mathrm{LR}}_{\mathrm{selected}}$** do**18:    $F\leftarrow ∥P_{y}-\sum_{i=1}^{U}\sigma_{v^{\left(i\right)}|H_{d_{i}}}^{2}-\sigma_{w}^{2}1_{M}∥$19:    **if** $F<F_{\min}$ **then**20:        $\hat{d}\leftarrow d$21:        $F_{\min}\leftarrow F$22:**return **$\hat{d}$

### 2.5. Computational Complexity Analysis

Table 1 represents the computational complexity of the proposed multiuser TO estimators. As seen, the proposed SMC estimator offers significantly lower computational complexity compared to the JMoM estimator at the expense of performance degradation in lock-in probability and MSE. It should be mentioned that the computational complexity of the JMoM and CA-JMoM can be reduced by employing dynamic programming methods, such as Viterbi algorithm.

### 2.6. Extension to Multiple Antennas

Let us now assume that the BS is equipped with $m_{r}$ receive antennas while the users have a single antenna. With the assumption of *U* independent single-input multiple-output (SIMO) channels, and independent and identically distributed (i.i.d) fading between the user antenna and the BS antennas, the JMoM multiuser TO estimation is formulated as

(25) $$\begin{array}{cc} {\hat{d}}_{1},\cdots ,{\hat{d}}_{U} & =\underset{-d_{\max}\leq d_{1},\cdots ,d_{U}\leq d_{\max}}{\mathrm{argmin}}\sum_{m=1}^{m_{r}}∥P_{y_{m}}-\sigma_{y|H_{d_{1},\cdots ,d_{U}}}^{2}∥\\ \; & =\underset{-d_{\max}\leq d_{1},\cdots ,d_{U}\leq d_{\max}}{\mathrm{argmin}}\sum_{m=1}^{m_{r}}∥P_{y_{m}}-\sum_{i=1}^{U}\sigma_{v^{\left(i\right)}|H_{d_{i}}}^{2}-\sigma_{w}^{2}1_{M}∥, \end{array}$$

where $P_{y_{m}}$ denotes the SAV of the received samples at the *m*th received antennas, and $\sigma_{v^{\left(i\right)}|H_{d_{i}}}^{2}$ is the sequence of the theoretical SoMs given hypothesis $H_{d_{i}}$, where the vector $\sigma_{v^{\left(i\right)}|H_{d_{i}}}^{2}$ is given in (23). Similarly, for the case of multiuser MIMO, we can write

(26) $$\begin{array}{cc} {\hat{d}}_{1},\cdots ,{\hat{d}}_{U} & =\underset{-d_{max}\leq d_{1},\cdots ,d_{U}\leq d_{max}}{\mathrm{argmin}}\sum_{m=1}^{m_{r}}∥P_{y_{m}}-\sigma_{y|H_{d_{1},\cdots ,d_{U}}}^{2}∥\\ \; & =\underset{-d_{\max}\leq d_{1},\cdots ,d_{U}\leq d_{\max}}{\mathrm{argmin}}\sum_{m=1}^{m_{r}}∥P_{y_{m}}-\sum_{i=1}^{U}m_{t_{i}}\sigma_{v^{\left(i\right)}|H_{d_{i}}}^{2}-\sigma_{w}^{2}1_{M}∥, \end{array}$$

where $m_{t_{i}}$ is the number of antennas at user $u_{i}$.

We can easily show that the computational complexity of the proposed JMoM TO estimator for the case of SIMO and MIMO is $O(Md_{\max}^{U}m_{r})$.

## 3. Simulations

In this section we first describe the default simulation setup parameters and then investigate the effect of each parameter on the performance of the proposed multiuser TO estimators by changing only one parameter at a time in each experiment.

### 3.1. Default Simulation Setup

Unless otherwise mentioned, the following simulation setup and parameters are set for the simulations. A ZP-OFDM system with 128-QAM modulation is considered. The channel is set up as a doubly selective Rayleigh multipath fading channel. The number of channel taps is $n_{h}=10$ and the channel taps are assumed to be uncorrelated in the delay domain. The maximum delay spread of the channel is set to be $\tau_{\max}=10$ μs. An exponential-decay function, i.e., $\sigma_{h_{l}}^{2}=\alpha \exp\left(-\beta l\right)$, $l=0,1,\ldots ,n_{h}-1$, where $p_{h}=\sum_{l=0}^{n_{h}-1}\sigma_{h_{l}}^{2}=1$, α=1/2.5244, and β=0.5, is considered to model the PDP of the doublyselective fading channel. A maximum Doppler spread of $f_{D}=2.880$ MHz is used for the Rayleigh fading channel which is equivalent to a speed of 35.75 m per second assuming the carrier frequency is 24.15 GHz. The channel taps for different users are set to be independent of each other.

The sampling time interval of the system at the receiver is set to $T_{\mathrm{sa}}=10^{-6}$s. In order to avoid ISI, the number of zero samples padded to each user’s signal is $n_{z}=15$. The number of data subcarriers, $n_{x}$, for both users is considered to be 128. The total data transmission power, i.e., the sum of the powers of the first and the second user, is set $\sigma_{x}^{2}=\sigma_{x_{1}}^{2}+\sigma_{x_{2}}^{2}=1$. The ratio of the user powers is defined as $c=\sigma_{x_{1}}^{2}/\sigma_{x_{2}}^{2}$. Unless otherwise mentioned, we set c=1. Moreover, a total number of 200 OFDM symbols are used for estimating the TO at the receiver.

The noise is considered to be AWGN and is set to be a zero-mean complex Gaussian random variable with variance $\sigma_{w}^{2}$. The variance of the noise is determined based on the SNR value, i.e., $E_{b}/N_{0}=\sigma_{x}^{2}p_{h}\log_{2}\left(M\right)/\sigma_{w}^{2}$ for 128-QAM moulation. The default SNR value, unless otherwise mentioned, is 5 dB. The TO of the users are modeled to be independent. Each user’s TO is an integer random variable that follows a discrete uniform distribution with in the range of $d_{1},\;d_{2}\in \left[-30,\;30\right]$. The performance of the proposed estimators is evaluated via $10^{4}$ Monte Carlo realizations for each scenario. For the CA version of the proposed estimators, a perfect parity check is considered.

### 3.2. Simulation Results

**Figure 1:**
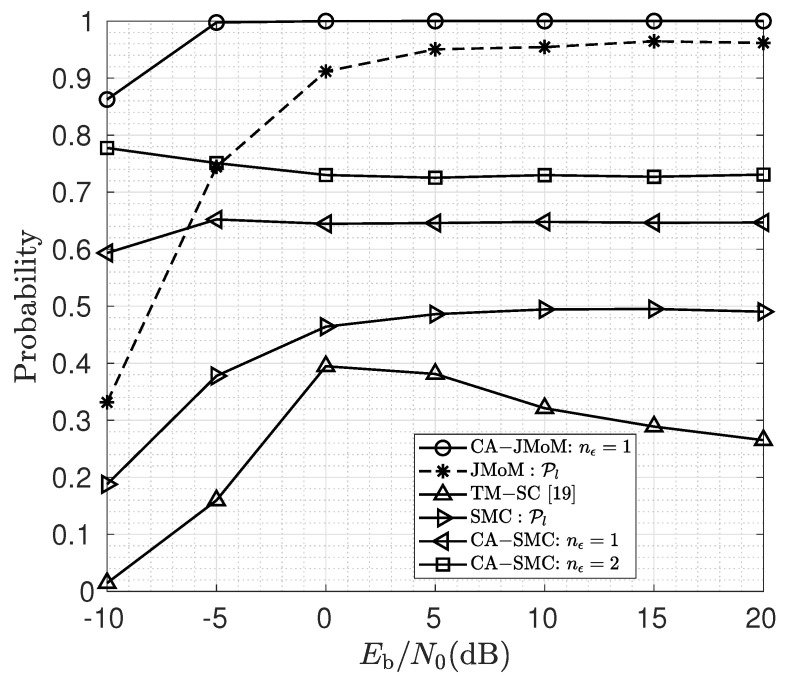
Lock-in probability of the proposed estimators for different values of $E_{b}/N_{0}$ (dB).

The lock-in probability of the proposed multiuser TO estimators for different values of $E_{b}/N_{0}$ is depicted in Figure 1. We have also shown the performance of the extended successive cancellation version of the TM estimator in [19], which we call it TM-SC. As seen, the proposed estimators significantly outperform the TM-SC estimator. The main reason is that the original TM estimator heavily relies on the noise-only samples which often do not exist in the multiuser scenarios. Since the performance of the TM-SC is poor, we will not report it in next figures. As seen, as the $E_{b}/N_{0}$ increases, the performance in terms of lock-in probability improves for the JMoM, the CA-JMoM, and the SMC estimators. More specifically, for the CA-JMoM estimator increasing $E_{b}/N_{0}$ from −10 to −5 dB increases the lock-in probability by more than 10%. For the jmom and the SMC algorithms, increasing $E_{b}/N_{0}$ from −10 to 5 increases the lock-in probability by about 60%. In order to explain this, without loss of generality, let us assume that $d_{2}\geq d_{1}$. When the signal of the second user arrives, if the $E_{b}/N_{0}$ is large enough, the jump in the variance of the samples thereafter would be large; hence, it is easier to distinguish the tos compared to when the $E_{b}/N_{0}$ is small. The lock-in probability of the CA-SMC algorithm remains relatively constant. Moreover, the CA version of the proposed estimators outperform their original ones. Given the very high lock-in probability for the CA-JMoM, one can decrease the number of OFDM samples used for estimation when the memory is limited.

**Figure 2:**
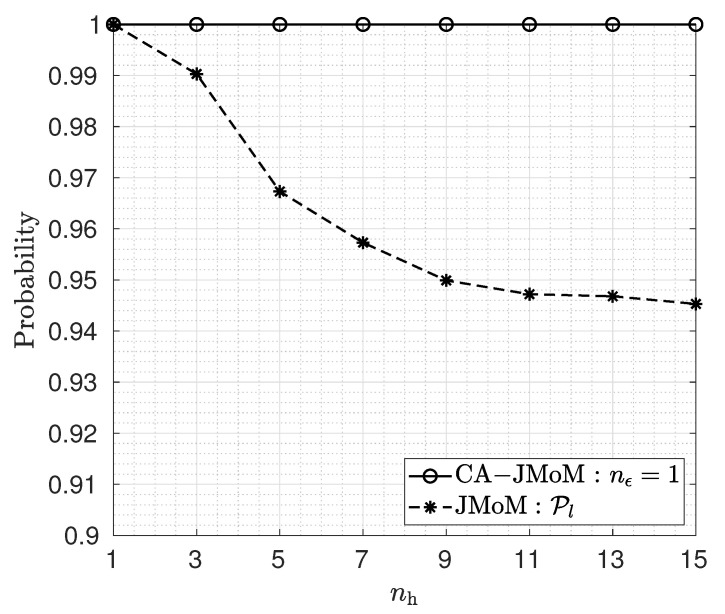
Lock-in probability for different values of number of channel taps when $n_{z}=10$.

The effect of the number of channel taps on the performance of the JMoM and the CA-JMoM estimators at 5 dB $E_{b}/N_{0}$ is depicted in Figure 2. As seen, the performance of the JMoM degrades as the number of channel taps increases. This is due to the fact that the number of noise-only samples decreases. Such samples play an important role in TO estimation. On the other hand, disregarding data samples and using noise-only samples for TO estimation results in poor performance. We also observe that the proposed CA-JMoM estimator can achieve significantly high lock-in probability.

**Figure 3:**
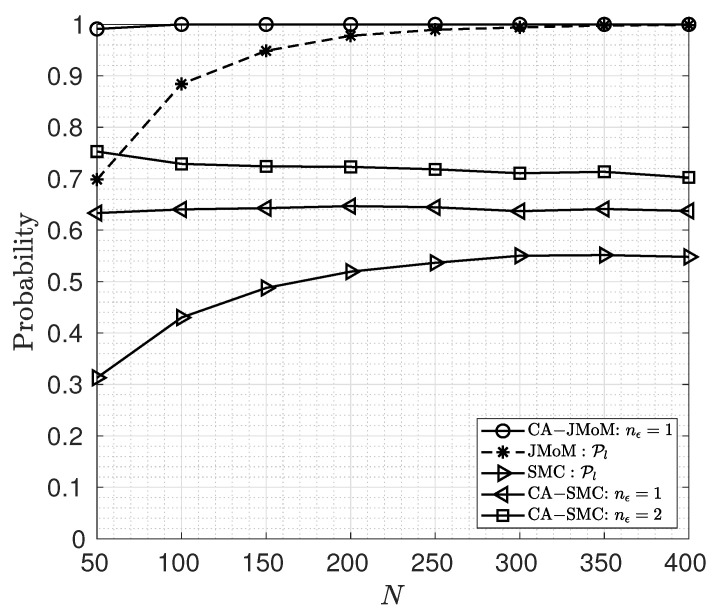
Lock-in probability for different values of OFDM symbols used for estimation.

The performance of the proposed TO estimators for different number of observation OFDM symbols is shown in Figure 3. As the number of observation symbols increases, the lock-in probability of the JMoM and the SMC estimators increases. However, the lock-in probability of the CA-JMoM and the CA-SMC estimators cannot reach lock-in probability of one. Hence, while the JMoM and the CA-JMoM are consistent estimators, their CA versions are inconsistent. As seen, the performance of the CA versions of the estimators does not change with *N* because perfect parity check is considered.

**Figure 4:**
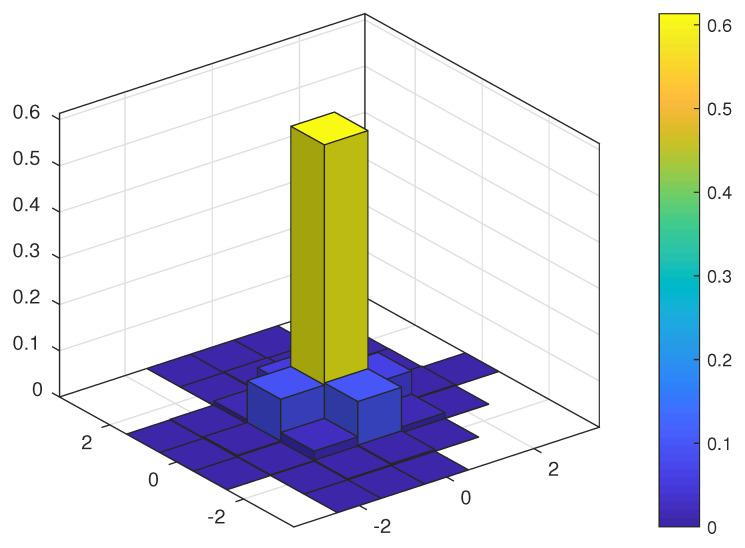
PMF of the TO estimation error for JMoM at $E_{b}/N_{0}=0$ dB.

**Figure 5:**
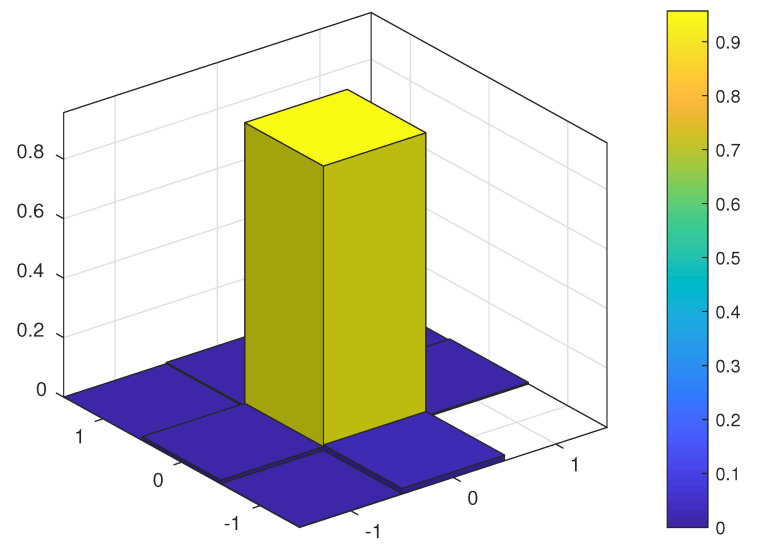
PMF of the TO estimation error for JMoM at $E_{b}/N_{0}=10$ dB.

**Figure 6:**
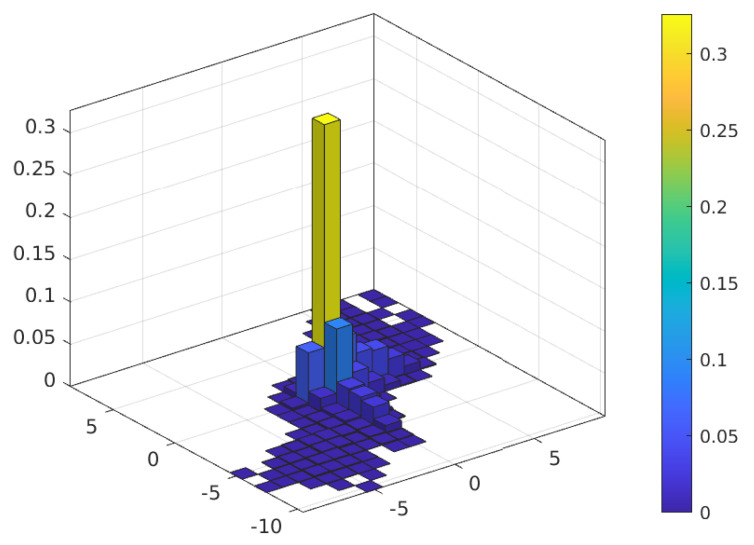
PMF of the TO estimation error for SMC at $E_{b}/N_{0}=10$ dB.

Figure 4 and Figure 5 show the probability mass function (PMF) of the TO estimation error of the JMoM estimator at 0 and 10 dB $E_{b}/N_{0}$, respectively. Figure 6 shows the PMF of the estimation error for the SMC estimator at 5 dB $E_{b}/N_{0}$. As seen, unlike an unbiased estimator, the PMF of the estimation error for the JMoM and SMC estimators is not symmetric around (0,0). However, the PMF becomes more symmetric as the number of observation OFDM symbols or $E_{b}/N_{0}$ increases. This can be seen in Figure 4 and Figure 5. Since the error is mostly concentrated around the actual TO value, i.e., error equal to (0, 0), for the JMoM (and relatively for the SMC), the CA-JMoM offers significantly high lock-in probability.

**Figure 7:**
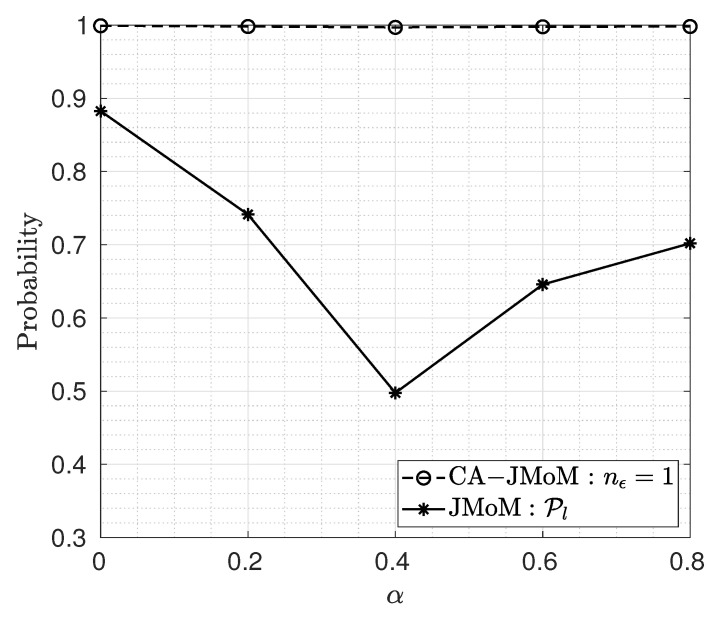
Lock-in probability for different coefficients in PDP estimation error.

Let us now study the effect of the PDP estimation error on the performance of the JMoM and the CA-JMoM TO estimators. As mentioned earlier, the PDP of the channel can be obtained through channel sounding prior to data transmission. However, there is PDP estimation error. Let us model the estimation error of the *k*-th channel tap as

(27) $$\begin{array}{c} {\hat{\sigma }}_{h_{k}}^{2}\in U[\left(1-\alpha \right)\sigma_{h_{k}}^{2},\left(1+\alpha \right)\sigma_{h_{k}}^{2}], \end{array}$$

where α∈[0,1], and $U[b_{1},b_{2}]$ denotes the continuous uniform distribution in the range of $\left[b_{1},b_{2}\right]$. The performance of the JMoM and the CA-JMoM estimators for different values of *a* is depicted in Figure 7. As seen, the performance of the JMoM estimator degrades in the presence of PDP estimation error. However, the CA-JMoM is robust to the PDP estimation error.

**Figure 8:**
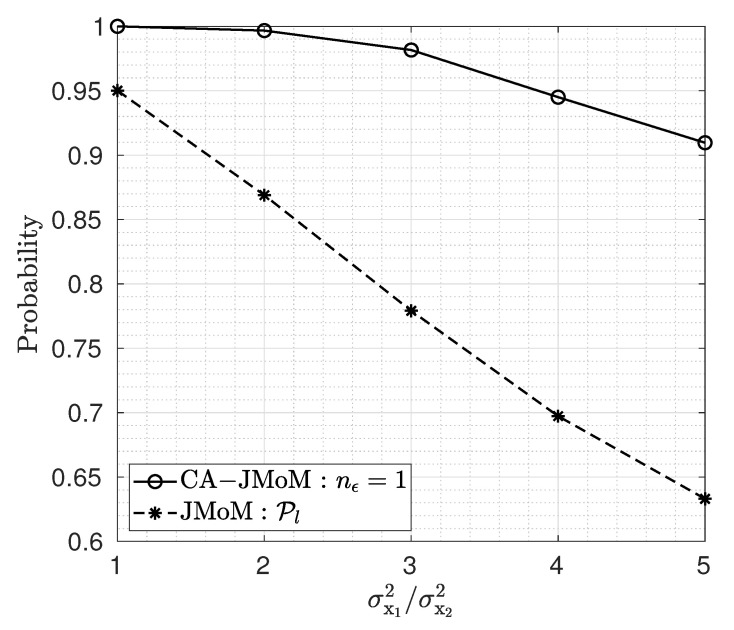
Lock-in probability versus power ratios of users.

The effect of the users’ power ratio, i.e., $c=\sigma_{x_{1}}^{2}/\sigma_{x_{2}}^{2}$, on the performance of the JMoM and the CA-JMoM estimator is shown in Figure 8. On the contrary to the SIC [28], the highest lock-in probability is achieved when the power is distributed equally between the users, i.e., c=1. The reason is that the signals in this situation are most distinguishable in terms of variance. As the gap between the users’ powers, increases, the signal with the lower power hides within the signal with a higher power, and hence, the performance degrades.

**Figure 9:**
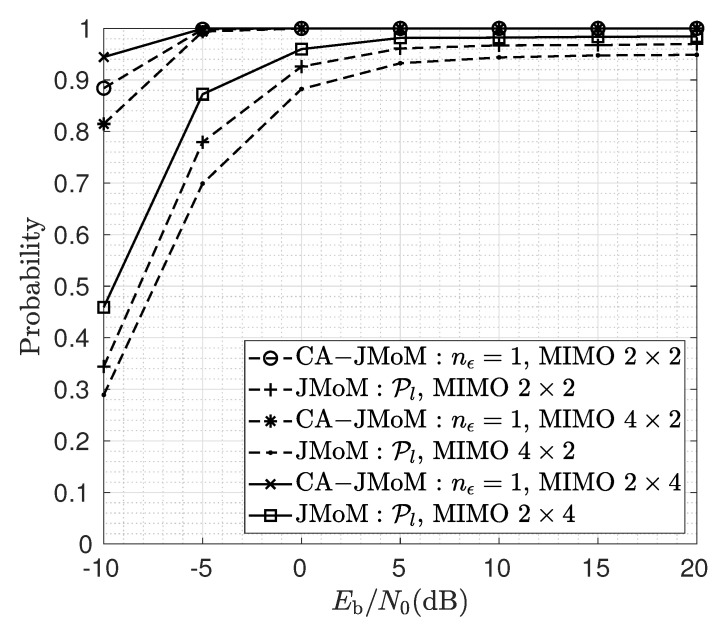
Lock-in probability for different number of transmit and receive antennas.

Figure 9 shows the performance of the JMoM and the CA-JMoM estimators for two users equipped with multiple transmit antennas. The BS also employs multiple receive antennas. The number of OFDM symbols used for estimation for 2×2, 2×4 and 4×2 multiuser MIMO scenarios, are 80, 60 and 60, respectively. As seen, the lock-in probability can be improved by employing multiple receive antennas. The higher number of antennas at the receiver achieves higher estimation accuracy because of the spatial diversity. On the other hand, the more number of antennas at the transmitter results in self-interference and thus performance degradation. The higher estimation accuracy for the multiple receive antennas allows a lower number of observation samples. We also observe that the gap between the JMoM and the CA-JMoM estimators decreases in the case of multiuser MIMO.

## 4. Conclusions

The problem of time synchronization in a multiuser uplink NOMA where users employ ZP-OFDM signals was investigated. We proposed two low-complexity NDA estimators, i.e., the JMoM and the SMC, for estimating the TO of the users. Moreover, the coding assisted version of the proposed estimators, i.e., the CA-JMoM and the CA-SMC, were developed for the case of short observation symbols. We also extended the proposed estimators to multiuser MIMO scenario. Existing NDA estimators [19,20] either have low lock-in probability, high computational complexity that prevents them from being employed in MIMO systems, or are designed for single-user scenarios. The proposed estimators in this paper address all of these issues. The lock-in probability of the proposed estimators was evaluated under various practical scenarios. Simulation results showed that the JMoM estimator offers high lock-in probability, and the CA-JMoM estimator can reach lock-in probability of one. Also, the highest lock-in probability for the JMoM and the CA-JMoM estimators is achieved when the power is distributed equally between the users. The future work is to further reduce the complexity of the JMoM and the CA-JMoM via dynamic programming techniques, such as Viterbi algorithm.

## Figures and Tables

**Table 1:** Complexity of the proposed estimators.

Metric	JMoM	SMC	CA-JMoM	CA-SMC
Complexity	$O(Md_{\max}^{U})$	$O(Md_{\max}U)$	$O(Md_{\max}^{U}$+$n_{\epsilon }^{U}M)$	$O(Md_{\max}$+$n_{\epsilon }^{U}M)$

## Data Availability

Not applicable.

## References

[1] Liu F., Cui Y., Masouros C., Xu J., Han T.X., Eldar Y.C., Buzzi S. (2022). Integrated Sensing and Communications: Towards Dual-functional Wireless Networks for 6G and Beyond. IEEE J. Sel. Areas Commun..

[2] Aydogdu C., Keskin M.F., Garcia N., Wymeersch H., Bliss D.W. (2019). RadChat: Spectrum Sharing for Automotive Radar Interference Mitigation. IEEE Trans. Intell. Transp. Syst..

[3] Dokhanchi S.H., Barreto A.N., Fettweis G. (2022). A Half-duplex Joint Communications and Sensing System using ZP-OFDM. Proceedings of the 2022 2nd IEEE International Symposium on Joint Communications & Sensing (JC&S).

[4] Liu Z., Quan Y., Wu Y., Xing M. (2022). Super-Resolution Range and Velocity Estimations for SFA-OFDM Radar. Remote Sens..

[5] Wang J., Wang P., Zhang R., Wu W. (2023). SDFnT-Based Parameter Estimation for OFDM Radar Systems with Intercarrier Interference. Sensors.

[6] Shi Q., Zhang T., Yu X., Liu X., Lee I. (2022). Time Domain IRCI-Free Pulse Compression for OQAM-OFDM Radar System. IEEE Syst. J..

[7] Noh H., Lee H., Yang H.J. (2022). ICI-Robust Transceiver Design for Integration of MIMO-OFDM Radar and MU-MIMO Communication. IEEE Trans. Veh. Technol..

[8] Pham T.M., Barreto A.N., Fettweis G.P. (2020). Efficient Communications for Overlapped Chirp-based Systems. IEEE Wirel. Commun. Lett..

[9] Lee S., Kim K., Lee K., Cho S., Choi S.U., Lee J., Koo B.T., Song H.J. (2022). An E-band CMOS Direct Conversion IQ Transmitter for Radar and Communication Applications. Proceedings of the 2022 IEEE Radio Frequency Integrated Circuits Symposium (RFIC).

[10] An S., Bu X., Wymeersch H., Zirath H., He Z.S. (2022). Millimeter-Wave Multi-Channel Backscatter Communication and Ranging with an FMCW Radar. Sensors.

[11] Muquet B., Wang Z., Giannakis G.B., de Courville M., Duhamel P. (2002). Cyclic Prefixing or Zero Padding for Wireless Multicarrier Transmissions?. IEEE Trans. Commun..

[12] Mashhadi M.B., Gündüz D. (2021). Pruning the Pilots: Deep Learning-based Pilot Design and Channel Estimation for MIMO-OFDM Systems. IEEE Trans. Wirel. Commun..

[13] Kang X.F., Liu Z.H., Yao M. (2022). Deep Learning for Joint Pilot Design and Channel Estimation in MIMO-OFDM Systems. Sensors.

[14] Zhao L., Guo W., Liu Y., Yang J., Wang W. Pilot Optimization for OFDM-based OTFS Systems over Doubly Selective Channels. Proceedings of the GLOBECOM 2020-2020 IEEE Global Communications Conference.

[15] Athisaya Anushya T., Laxmikandan T., Manimekalai T. (2022). Zero Padded Dual Index Trimode OFDM-IM. Proceedings of the 2022 International Conference on Wireless Communications Signal Processing and Networking (WiSPNET).

[16] Neelam S.G., Sahu P. (2022). Digital Compensation of IQ Imbalance, DC Offset for Zero-Padded OTFS Systems. IEEE Commun. Lett..

[17] Thaj T., Viterbo E. (2020). Low Complexity Iterative Rake Decision Feedback Equalizer for Zero-padded OTFS Systems. IEEE Trans. Veh. Technol..

[18] Karpovich P., Zielinski T.P. (2022). Random-Padded OTFS Modulation for Joint Communication and Radar/Sensing Systems. Proceedings of the 2022 23rd International Radar Symposium (IRS).

[19] Le Nir V., van Waterschoot T., Duplicy J., Moonen M. (2010). Blind Coarse Timing Offset Estimation for CP-OFDM and ZP-OFDM Transmission over Frequency Selective Channels. EURASIP J. Wirel. Commun. Netw..

[20] Roshandeh K.P., Mohammadkarimi M., Ardakani M. (2021). Maximum Likelihood Time Synchronization for Zero-Padded OFDM. IEEE Trans. Signal Process..

[21] Roshandeh K.P., Mohammadkarimi M., Ardakani M. (2023). Low Complexity Time Synchronization for Zero-padding based Waveforms. arXiv.

[22] Ahmed M., Khan W.U., Ihsan A., Li X., Li J., Tsiftsis T.A. (2022). Backscatter Sensors Communication for 6G Low-powered NOMA-enabled IoT Networks under Imperfect SIC. IEEE Syst. J..

[23] Ihsan A., Chen W., Asif M., Khan W.U., Wu Q., Li J. (2022). Energy-efficient IRS-aided NOMA beamforming for 6G Wireless Communications. IEEE Trans. Green Commun. Netw..

[24] Liu Y., Zhang S., Mu X., Ding Z., Schober R., Al-Dhahir N., Hossain E., Shen X. (2022). Evolution of NOMA toward Next Generation Multiple Access (NGMA) for 6G. IEEE J. Sel. Areas Commun..

[25] Oleiwi H.W., Al-Raweshidy H. (2022). Cooperative SWIPT THz-NOMA/6G Performance Analysis. Electronics.

[26] Morelli M., Kuo C.C.J., Pun M.O. (2007). Synchronization techniques for orthogonal frequency division multiple access (OFDMA): A tutorial review. Wirel. Commun. Mob. Comput..

[27] Jiang T., Wu Y. (2008). An overview: Peak-to-average Power Ratio Reduction Techniques for OFDM Signals. IEEE Trans. Broadcast..

[28] Cheng H., Xia Y., Huang Y., Yang L. (2022). Improper Gaussian Signaling for Downlink NOMA Systems With Imperfect Successive Interference Cancellation. IEEE Trans. Wirel. Commun..

[29] Fanari L., Iradier E., Bilbao I., Cabrera R., Montalban J., Angueira P., Seijo O., Val I. (2022). A Survey on FEC Techniques for Industrial Wireless Communications. IEEE Open J. Ind. Electron. Soc..

